# Spatial Control of Functional Response in 4D-Printed Active Metallic Structures

**DOI:** 10.1038/srep46707

**Published:** 2017-04-21

**Authors:** Ji Ma, Brian Franco, Gustavo Tapia, Kubra Karayagiz, Luke Johnson, Jun Liu, Raymundo Arroyave, Ibrahim Karaman, Alaa Elwany

**Affiliations:** 1 Department of Materials Science and Engineering, Texas A&M University, College Station TX, 77843, USA; 2 Department of Industrial and Systems Engineering, Texas A&M University, College Station TX, 77843, USA.

## Abstract

We demonstrate a method to achieve local control of 3-dimensional thermal history in a metallic alloy, which resulted in designed spatial variations in its functional response. A nickel-titanium shape memory alloy part was created with multiple shape-recovery stages activated at different temperatures using the selective laser melting technique. The multi-stage transformation originates from differences in thermal history, and thus the precipitate structure, at various locations created from controlled variations in the hatch distance within the same part. This is a first example of precision location-dependent control of thermal history in alloys beyond the surface, and utilizes additive manufacturing techniques as a tool to create materials with novel functional response that is difficult to achieve through conventional methods.

The properties of metallic materials are generally very sensitive to their thermal history, and for many engineering alloys, sophisticated processing techniques have been developed to provide a range of desired properties by precisely manipulating the thermal history of the part. However, complex engineering applications in medicine^1^ and aerospace^2^ often require materials with properties that not only change from part to part, but at various spatial locations within the same part. This need cannot be satisfied with conventional processing beyond the material’s surface. In the present work, we demonstrate a method of creating programmed location-dependent functional properties in a shape memory alloy through the control of local thermal history with an additive manufacturing technique.

Additive manufacturing (AM), or 3D Printing, has already achieved significant advancements since its inception in the early 1980s, and led to a revolution in the design and manufacture of parts in traditional 3D space. Now it is witnessing further evolution through the introduction of 4D printing. The fourth “D” refers to the dimension of time, where the 3D printed part continues to undergo a controlled change in shape or properties in response to an external stimulus over time such as heat, stress, light, and moisture, among others^3^,^4^,^5^,^6^. While the majority of this approach is found in polymers, 4-D printing of active metallic materials have been shown in nickel-titanium based shape memory alloys (SMAs)^7^,^8^,^9^,^10^,^11^,^12^. The physical and functional responses of the alloy depend greatly on the processing schemes employed during fabrication. For example, in powder-bed based AM methods, important processing parameters include energy source power and scan speed, layer density, and scan line spacing among many others. Variations in these parameters result in corresponding variations in the maximum shape change (transformation strain), temperature interval at which shape change takes place (transformation temperatures), and mechanical strength^7^,^8^,^9^,^10^,^11^,^12^. These functional changes correlate with changes in the microstructure of the alloy caused by differences in the thermal history within the fabricated part during processing that are driven by different sets of processing parameters. Based on this knowledge, we address the inverse problem: by controlling the processing parameters, SLM-fabricated Ni-Ti SMAs can be tailored to achieve spatial and time-dependent functional responses.

## Results and Discussions

**Figure 1:**
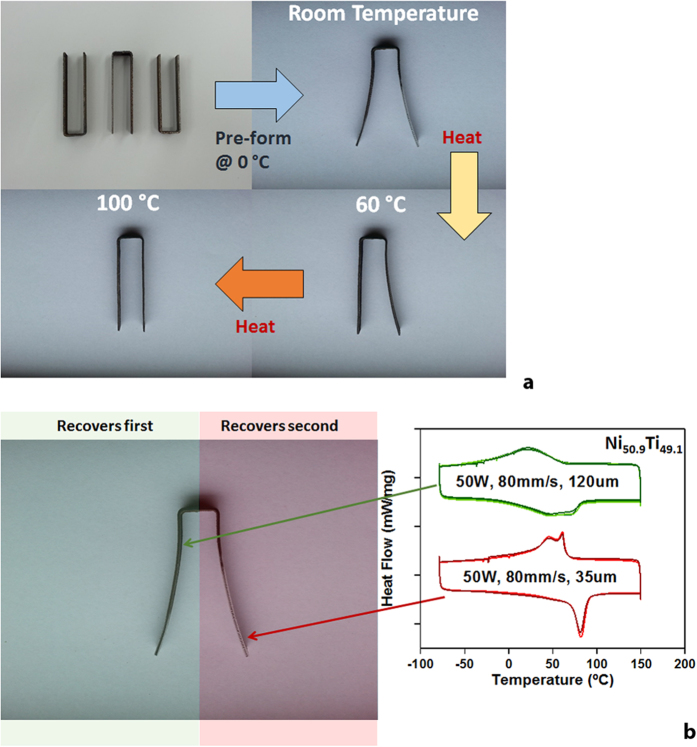
(**a**) Multi-stage shape recovery in a U-shaped additively manufactured NiTi build piece using Selective Laser Melting. Two “arms” of the piece activate their shape recovery at different temperatures, creating a location-dependent active response; (**b**) The location-dependent active response is created by changing the SLM processing parameters (shown in the DSC curves on the right) at different sections of the build, which results in differences in the transformation temperatures in corresponding sections.

Two different sets of SLM processing parameters were used to process two different sections in a single Ni_50.9_Ti_49.1_ SMA U-shaped build piece. The two arms of the U-shape build piece were subsequently pre-formed at 0 °C in the martensite state. As the part is heated, the reverse transformation from martensite to austenite occurs, resulting in a two-stage shape change where different sections of the part activate the shape memory response at different temperature intervals and at different rates. The transformation is illustrated in Fig. 1a.

The question still remains as to which parameter was varied between the arms to create this two-stage transformation in one single build piece. A commonly used metric in the characterization of metallic AM techniques is the energy density (E_d_), expressed as:


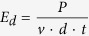


where *P* is the laser (or electron)-beam power, *v* is the scan velocity, *d* is the hatch distance (distance between successive passes of the beam within the same layer), and *t* is the layer thickness. Energy density is the amount of energy deposited into a unit volume of the material during processing, and is used as a metric for the thermal energy available to the material as it evolves from the powder to the solid state. During the build, the energy density of the right arm was made 3 times larger than that of the left arm through a 3-fold reduction of hatch distance (120 μm vs 35 μm, see methods for details), while the other processing parameters remained identical. The lower energy density process used on the left arm results in a lower transformation temperature and shape recovery begins at roughly 0 °C, as shown in the differential scanning calorimetry curve (DSC) in Fig. 1b. In contrast, shape recovery in the right arm, which was processed at a higher energy density, does not occur until 60 °C, which is after the completion of the shape recovery in the left arm. As such, shape recovery in the material occurs in two distinct steps. This capability offers flexibility in the design of active and deployable devices, making it easier to create tailored or programmable deployments and enabling multi-shape active systems.

By spatially changing the SLM process parameters, it is possible to vary the amount of thermal energy deposited per unit volume of material, which results in position-dependent thermal histories which in turn create spatial variation in the microstructure. In the specific case illustrated in Fig. 1, the two-stage functional response is derived from two different sets of hatch distances used during fabrication. On the other hand, materials with complex microstructures and functional responses are inadequately described by using only the deposited energy density. It has been shown that the transformation temperature can vary greatly following changes in the individual parameters such as beam power or scan velocity^9^, even if the overall energy density is kept constant (See Figure S1 in the supplementary information). This is because any material point in the built part is subject to a rather complex thermal history comprising multiple re-melting, solidification and re-heating cycles. Characterizing the processing conditions in terms of energy density, while useful, is a significant simplification. Since thermal history of a material controls the microstructure and the properties of the material, there is an immediate need to understand the role of process parameters on the thermal history of a given location in 3D printed parts and to monitor the thermal histories.

**Figure 2:**
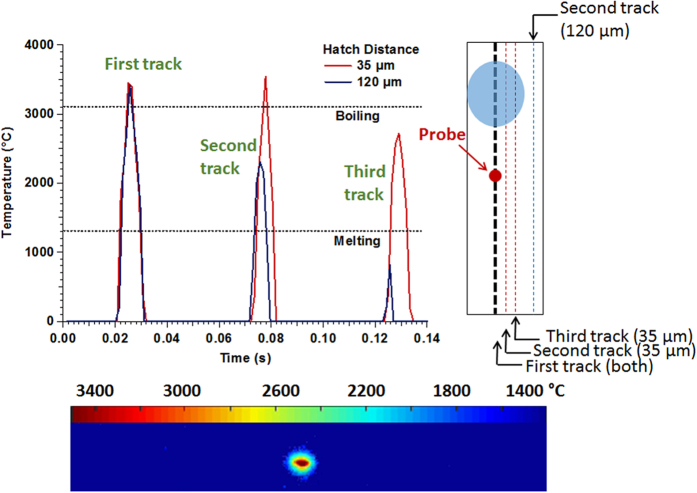
The time-temperature plot shows the temperature history of a probe point during the initial and two adjacent laser passes as recorded by a pyrometer. The sample built with 120 μm hatch distance experiences lower temperatures during adjacent laser passes. This is caused by the larger distance between adjacent tracks in the 120 μm hatch distance sample. The melt pool (bottom figure from a pyrometer capture, and illustrated as the circled area on the schematic on the right) from adjacent tracks in the 35 μm hatch distance sample experiences significant overlap, resulting in repeated re-melting and reheating.

To better understand the impact of SLM processing protocol on location-dependent thermal histories, a two-wavelength pyrometer is used to characterize the spatial thermal history of two model builds, with each model corresponding to one of the hatch distances used to make the U-shaped part; this is illustrated in Fig. 2. Each model build consists of three parallel scan tracks with the corresponding hatch spacing, and the thermal history is experimentally recorded at a given point in the first scan line as the laser completes the three parallel tracks. A representative post-processed thermal image from the pyrometer can be seen in Fig. 2 (bottom image with temperature color mapping) along with the entire thermal history of one fixed point on the surface (top chart in Fig. 2). The melt pool is approximately 205 μm in width and 250 μm in length independent of hatch distance. As the laser moves to an adjacent track, the material in the original track is re-melted since the size of the melt pool is greater than the spacing between the tracks. However, re-melting occurs many more times in the 35 μm hatch distance section than the 120 μm hatch distance section. The first peak in the thermal history (top chart in Fig. 2) corresponds to the temperature when the laser moves directly through the probe location, whereas the second and the third peaks correspond to the two subsequent laser passes in the adjacent tracks. Differences in the thermal peaks corresponding to each hatch distance illustrate the corresponding differences between the temperature histories of the two parameters sets: while the 35 μm hatch distance sample continues to undergo re-melting after the third pass, the temperature in the third pass for the 120 μm hatch distance sample is no longer sufficient to reach the melting temperature.

As layers are added, the power source will pass over the solidified area, causing further reheating and re-melting. Due to the nature of SLM as a powder-bed AM process, the pyrometer is not able to capture temperature information beyond the top layer of the build. Instead, here, a finite-element model was constructed in COMSOL Multiphysics^®^ software to study the effects of hatch distance on the three-dimensional thermal history. The model accounted for several physical phenomena associated with SLM processes including conduction, convection, radiation, phase transitions, and evaporation. The temperature dependent thermo-physical properties of Ni-Ti were also taken into consideration. Using this model, the deposition of 3-tracks on a solid substrate was simulated for both hatch distances (35 μm and 120 μm).The predicted melt pool size and depth from the COMSOL model were validated using the experimental measurements of the melt pool size. Using this validated model, a simulation was then performed for the same laser beam trajectory as the experiment performed with the pyrometer. As the beam moves along its build path, the melt pool is carried along producing re-melting both in the current surface layer and the previously deposited layers underneath it. This is demonstrated by the temperature-time diagram (Fig. 2), which follows a single point along the first track on the surface layer. As each additional layer is added, the same scan tracks are repeated, and a spike in the temperature is observed at the original point in the first layer.

**Figure 3:**
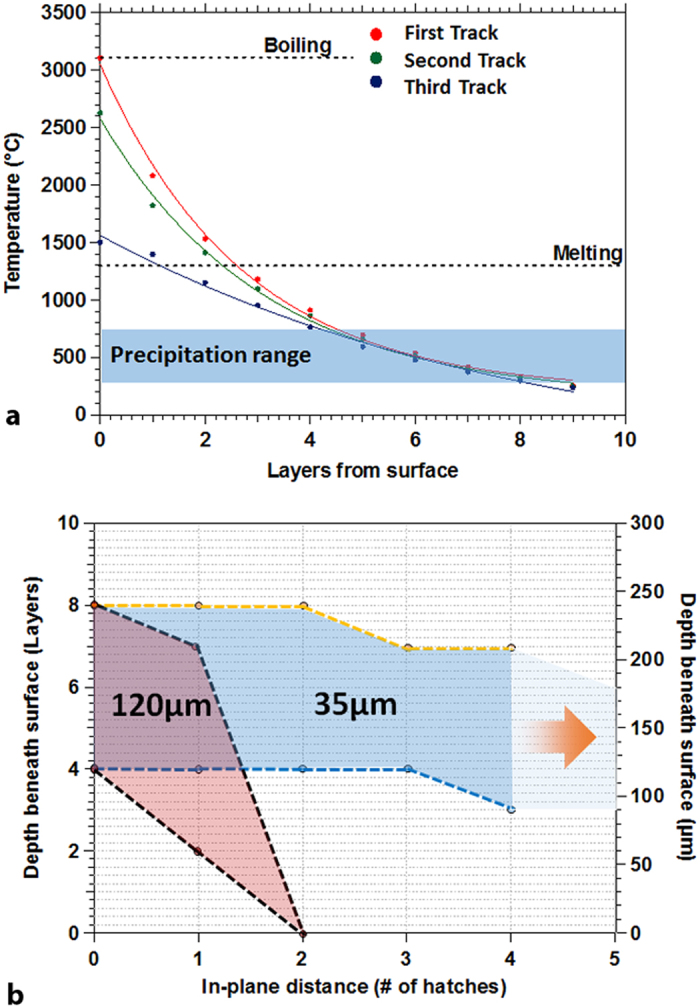
(**a**) Simulated temperature history of a probe point in a build as additional layers above it are created for a 35 μm hatch distance sample. The curves show the history when the laser is directly above the point or when it is one hatch spacing (35 μm) or two hatch spacing (70 μm) away; (**b**) boundary of the locations, as compared to the surface, for which the temperatures are suitable for precipitation nucleation and growth for samples of hatch distances of 35 μm and 120 μm.

Figure 3a shows the predicted temperature histories of layers underneath the surface for the 35 μm hatch distance sample: while the first three layers result in high enough temperature to re-melt the material, latter passes result only in re-heating. Similarly, re-melting occurs in one or two layers beneath the surface for the pass directly adjacent to the probe point, and only re-heating occurs when the laser is two track spacing away. After three additional layers have been built above any given point in the part, this location of the material no longer melts, but instead experiences a very complex cycle of re-heating and cooling. When a wider hatch distance is used instead, the material reaches a permanently solid state much sooner, and experiences a longer duration of the reheating process.

**Figure 4:**
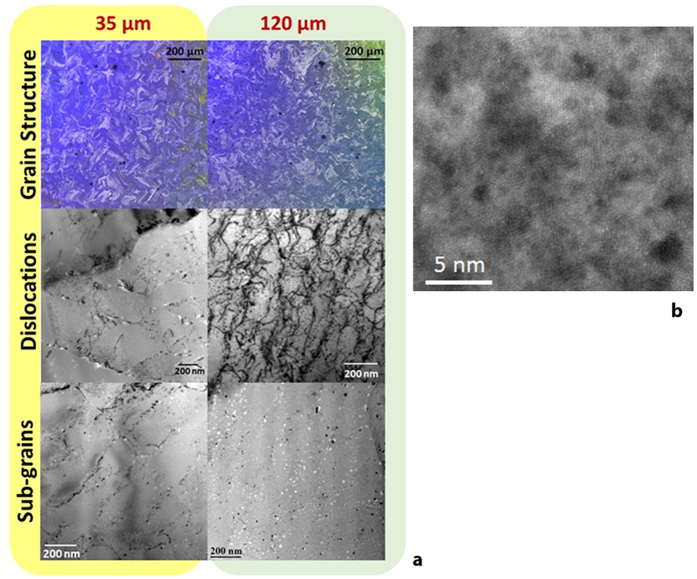
(**a**) Optical and TEM micrographs of the 35 μm and 120 μm hatch distance samples, illustrating differences in the grain size and structure, dislocation density, and second phase and sub-grain structures; (**b**) high resolution image of the 120 μm hatch distance sample showing small precipitates approximate 1–3 nm in size.

Microstructures of the as-fabricated samples are illustrated in Fig. 4 for both the 35 μm and 120 μm hatch distance samples. The morphology of the grains in both samples are narrow and elongated with size on the order of 100 μm, and does not show signs of columnar arrangement when viewed from the side surface. Similar grain morphologies have been reported in both NiTi^8^ and Co-Cr-Mo^13^ at low laser power and slow speed which results in small thermal gradient. While the checkerboard pattern is more clearly visible in the case of the 35 μm hatch distance sample compared to the 120 μm hatch distance sample, there does not appear to be a significant difference in the grain size that would contribute to differences in transformation temperatures. On the other hand, TEM micrographs reveals that the dislocation density appears to be higher in the 120 μm hatch distance samples. The likely explanation is that the 35 μm hatch distance sample experiences a greater number of remelting and reheating cycles as illustrated in Fig. 3, allowing for sufficient time for dislocation recovery to take place. A higher density of dislocations, which likely formed in the austenite during rapid solidification, will result in a reduction in the transformation temperatures^14^,^15^,^16^. Since the dislocation density of the 120 μm hatch distance sample appears to be higher than that of the 35 μm hatch distance case, it is also expected that the transformation temperatures would be comparatively lower in the 120 μm hatch distance samples which indeed match our observation. However, the dislocation density does not appear to be very high, and it is not clear if this difference alone is sufficient to explain the variations in the transformation temperatures.

In addition to dislocations, TEM micrographs reveal second phase particles at two different lengths scale. Larger second phases roughly 10–20 nm in size are observed with high density inside the matrix of both the 35 μm and 120 μm hatch distance samples. These particles are arranged in a cell-like structure that appears to delineate sub-grain boundaries within the matrix. Energy Dispersive Spectrometry (EDS) measurements of these particles indicate that they are Ti_2_Ni. This is somewhat surprising since the overall composition of the material remains nickel-rich even after SLM fabrication. However, it is known that the formation kinetics of the Ti_2_Ni phase is very fast and it is known to appear even in Ni-rich compositions from oxygen stabilization or composition gradients^17^,^18^. Indeed, a small number of such particles appears to contain oxygen, but the low composition of oxygen does not appear sufficient to form the well-known Ti_4_Ni_2_O type oxide. It is possible that the rapid solidification does not allow sufficient time for a fully formed oxide to appear and a pre-cursor type region is created instead. Nevertheless, there does not appear to be a clear difference between the Ti_2_Ni distribution between the 35 μm and 120 μm hatch distance samples. A second class of second phase particles approximately 1–3 nm were observed in the background of the matrix (Fig. 4b). EDS results show that the non-Ti_2_Ni regions, which contains both the true matrix and the small precipitates, are nickel rich, which suggests that the small precipitates may be nickel-rich as well. Due to the small size and difficulty in imaging the samples caused by the martensitic structure, composition and structure of such precipitates could not be determined. To better understand the nature of such precipitates, we utilize a complementary computational approach.

For nickel-rich Ni-Ti compositions, it is known that a series of Ni-rich precipitates are formed as the sample is aged at temperatures between approximately 200–700 °C due to the metastable nature of the B2 austenite. The sequence of formation for these precipitates follows the Ni_4_Ti_3_ → Ni_3_Ti_2_ → Ni_3_Ti sequence, with Ni_4_Ti_3_ and Ni_3_Ti_2_ being metastable compounds^19^. The formation and coarsening of these precipitates has profound influences on the functional response of the Ni-Ti alloy, in particular the transformation temperature since they change the composition of the matrix material. In fact the change in the matrix composition about one atomic percent nickel alters the transformation temperatures by over 200 °C^20^. In the samples with no precipitates, transformation temperatures of nickel-rich Ni-Ti SMAs are far below room temperature. However, formation of the aforementioned Ni-rich precipitates extracts the excess nickel from the matrix, and causes transformation temperatures to rise. Additionally, formation of these precipitates may also cause barriers that impede propagation of the reversible phase transformation front. The combined effect of composition and barriers to transformation makes precipitates a primary driver of transformation temperatures in Ni-rich Ni-Ti SMAs, and change in the precipitate structure is immediately reflected in a change in the functional response.

The re-melting-cooling-reheating cycles shown in Fig. 1b (experimental measurements) and 3.a (model predictions) that take place during SLM cause the part to pass through the temperature region where precipitate formation occurs on multiple occasions. In some cases, a subsequent re-heating cycle may also bring the temperature above into the single-phase region where precipitates that have already formed may then dissolve away. Based on the thermal histories shown in Figs 2 and 3a and considering the temperature range for precipitation nucleation and coarsening, we construct a work envelope that shows the region of fabrication where precipitate formation is expected for the 35 μm and 120 μm hatch distance samples (Fig. 3b). For the 35 μm sample, the precipitation is expected at any location approximately between 3 and 8 layers, or 100–250 μm beneath the working surface when the laser is directly above the probe point or at least within 5 hatch spacing. In contrast, the region of precipitate activity in the 120 μm sample is restricted to approximately 4–7 layers (120–200 μm) beneath the working surface for up to two adjacent hatch spacing. Since the velocity of the laser is held constant, this means that the 35 μm sample stays in a temperature region for precipitation activity for a longer time than the 120 μm sample, and is expected to form a larger volume fraction of precipitates.

In order to understand the effect of hatch distance on the precipitate evolution throughout the fabricated parts, we developed a computational framework from the COMSOL model described above, with predicted thermal histories, in conjunction with a precipitation evolution model that incorporates nucleation, growth and coarsening using a Numerical Kampmann Wagner approach^21^ in the MatCalc precipitation modeling software^22^,^23^. With the predicted thermal histories, the precipitation model can predict the volume fraction of the precipitates at a given location in the fabricated parts. Based on the simulation performed in MatCalc for the nucleation and growth of the Ni_4_Ti_3_ precipitates (Figure S2), the predicted thermal history in Fig. 3a yields a higher volume fraction of precipitates in the 35 μm hatch distance sample at depth greater than 60 μm, or 2 layers, beneath the surface as compared to the precipitates in the 120 μm hatch distance sample. The DSC results in Fig. 1 show that a higher transformation temperature is obtained in the sample with smaller hatch distance. Therefore, this can be interpreted in light of the qualitatively predicted effect of the complex thermal history on the precipitation behavior in the Ni-rich Ni-Ti SMA as the result of increased Ni-depletion (with a subsequent increase in transformation temperature) in the matrix due to higher volume fraction of the precipitates.

The results discussed so far illustrate how the dramatic sensitivity of the shape memory response of Ni-Ti SMAs to microstructure–i.e. dislocation density or precipitate distribution–can be exploited by any approach that provides the ability to control microstructure via control of process parameters. As already shown, manipulation of process parameters during AM can result in controlled spatial variation of thermal histories that can be mapped to control of microstructural features and properties. In the case of Ni-Ti SMAs, this degree of microstructural/functional control implies the possibility of tailored, location-dependent multi-stage shape recovery, enabling the fabrication of complex, deployable structures. We demonstrate this through the design of a hand shown in the supplementary material (Figure S3). Further development of this technology can potentially generate many novel solutions to technological challenges requiring fine control of functional behavior in parts with complex geometries, i.e. monolithic machines.

In broader terms, the ability to fine-tune material properties based on location allows a marriage between a design of form and function: engineers and designers no longer need to compromise in design in order to accommodate existing materials. Compared to other manufacturing techniques, AM is thus uniquely suited to expand the design envelope whereby the boundaries between materials, process and part design are effectively blurred. With freedom, however, come important challenges as the degree of complexity of the design process requires the solution of complex, coupled inverse problems connecting form and function to microstructure and ultimately to processing parameters. A full experimental exploration and exploitation of this enormous design space is prohibitive and computer-aided approaches, in the spirit of the Integrated Computational Materials Engineering (ICME) framework may be used to at least constraint the space to be explored. The forward process-microstructure connections built, thanks to the coupling of physics-based thermal and microstructure evolution models, the initial steps in this general direction although design necessitates the solution of the inverse problem connecting desired function to microstructure and ultimately processing conditions (and materials chemistry).

In summary, here we demonstrate a first example of AM-fabricated functional metal alloy with controlled spatial variations in its stimulus-response characteristics, i.e. 4-D printed functional alloy, caused by location-dependent thermal history created from rational variation of the processing parameters during fabrication. We also show the feasibility of using metallic AM techniques as a tool to design materials with unique properties bespoke for a specific application.

## Experimental Methods

A commercial Ni_50.9_Ti_49.1_ (at.%) rod (SAES-Getter) was gas atomized (Nanoval GmbH) to produce a powder with a D50 size of 18.5 μm. The powder was consolidated using a Phenix (3D Systems) PXS laser melting system using at laser power of 50 W, laser speed of 80 mm/s, layer thickness of 30 μm, and hatch distance of 35 μm or 120 μm. A straight-line scanning strategy was used where the laser reversed its traveling direction for each successive scan track. After each layer, the direction of the laser tracks was rotated 90 degrees. The wall thickness of the U-shapes were 0.75 mm, and its length 50 mm, and the direction of the laser travel is oriented at a 45° angle with respect to the wall of the U-shapes. The samples were built onto Ni_50_Ti_50_ substrates at room temperature under flowing ultra-high purity argon. After fabrication, specimens were removed from the substrate using wire electrical discharge machining (EDM). DSC experiments were performed in a TA instruments Q2000 under flowing nitrogen. The 2.5 × 2.5 × 1 mm samples heated and cooled at a rate of 10 °C/s. Optical microscopy were conducted on samples prepared by mechanical polishing with a final step of 0.05 μm colloidal silica, followed by electropolishing in 3 M H_2_SO_4_ in 1:1 ethanol–methanol solution at 20 °C and voltage of 20 V^8^. A final color-etching step is then conducted in a solution containing 120 mL deionized water, 15 mL 1 M HCl (Sigma-Aldrich), 15 g Na_2_S_2_O_5_ (Sigma-Aldrich), 10 g K_2_S_2_O_5_ (Sigma-Aldrich), and 2 g NH_4_HF (Alfa-Aesar) following the work of Bormann *et al*.^8^. Optical micrographs were acquired on a Keyence VHX-1000 microscope with polarized light. Transmission electron microscopy (TEM) samples were prepared through focused ion beam machining on a Tescan LYRA-3 Focused Ion Beam Microscope. Pre-thinned 15 μm × 15 μm × 1 μm slides are prepared under 30 kV gallium ion beam. The sample is then lift out, welded to a holder, and then thinned under decreasing accelerating voltage from 30 kV to 5 kV to reduce the ion damage until it became transparent (<100 nm) under 5 kV electron beam for SEM imaging. TEM imaging was performed on a FEI Tecnai G2 F20 ST FE-TEM equipped with an EDAX instruments EDS detector at room temperature.

A three dimensional finite-element model consisting of a thin layer of powder and a thick substrate with respective dimensions of 4.5 × 0.5 × 0.03 mm and 4.5 × 0.5 × 0.3 mm was created using the COSMOL Multiphysics^®^ software. NiTi was selected as the material for both the powder bed and the substrate. The powder layer was modeled as a continuum media with initial porosity of 0.35. The proposed heat transfer model accounted for several physical phenomena associated with complex SLM processes including conduction, convection, radiation, phase transitions, and evaporation. The laser beam was defined as a moving surface heat source with Gaussian distribution. The temperature dependent thermo-physical properties of NiTi were incorporated, which is essential for an accurate prediction of the thermal history. Since the powder layer was assumed as continuum, the effective thermo-physical properties for the powder material were also considered.

The SLM system was custom instrumented with a thermal imaging sensor to experimentally measure the temperature using high speed thermography as a validation for the COSMOL model. The sensor used is a ThermaViz^®^ developed by Stratonics Inc, and is a dual-wavelength pyrometer with CMOS imaging technology and 1300 × 1000 pixels field of view (FOV) mapped to a 26 × 20 mm spatial resolution, yielding 20 μm per pixel. The pyrometer captures frames at a rate of 100 Hz for the full FOV, and can achieve up to 1 kHz frame rates for partial FOV.

## Additional Information

**How to cite this article:** Ma, J. *et al*. Spatial control of functional response in 4D-printed active metallic structures. *Sci. Rep.* **7**, 46707; doi: 10.1038/srep46707 (2017).

**Publisher's note:** Springer Nature remains neutral with regard to jurisdictional claims in published maps and institutional affiliations.

## Supplementary Material

Supplementary Information
