# Supplementary material for: Spatial Control of Functional Response in 4D-Printed Active Metallic Structures
Source: Sci Rep. 2017 Apr 21;7:46707. doi: 10.1038/srep46707 (PMC5399446; doi:10.1038/srep46707)
Supplement: Supplementary Information [file srep46707-s1.pdf]

Supplementary information for:

Spatial Control of Functional Response in 4D-Printed Active Metallic Structures

Ji Ma[1,*], Brian Franco[1], Gustavo Tapia[2], Kubra Karayagiz[1], Luke Johnson[1], Jun Liu[1], Raymundo Arroyave[1], Ibrahim Karaman[1], Alaa Elwany[2]

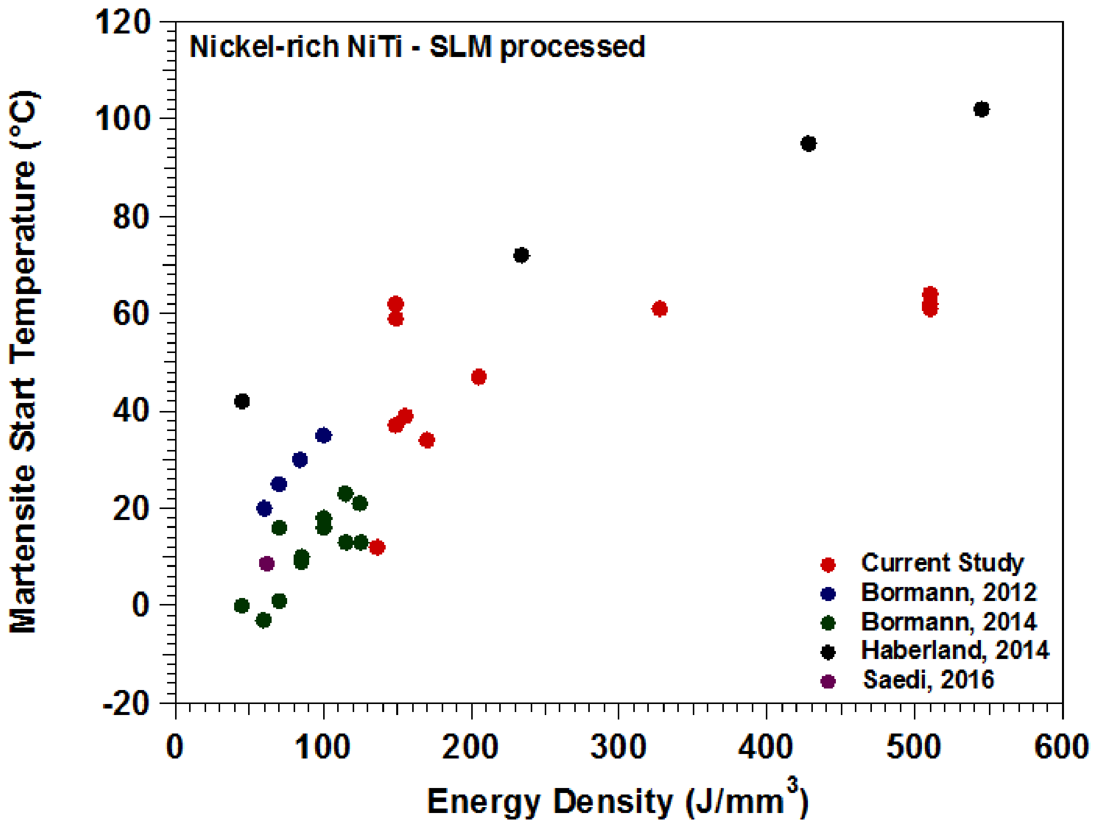

Figure S1: Summary of the effect of energy density on the martensite start temperatures of nickel-rich (50.7-50.9% Ni) Ni-Ti shape memory alloy fabricated using the selective laser melting process.

References for Figure:

[Bormann, 2012] Bormann T, Schumacher R, Mueller B, Mertmann M, de Wild M, Müller B. *Journal of Materials Engineering and Performance*, **21**, 2519-2524 (2012).

[Bormann, 2014] Bormann T, Mueller B, Schinhammer M, Kessler A, Thalmann P, Müller B, de Wild M. Materials Characterization 94, 189-202 (2014).

[Haberland, 2014] Haberland C, Elahinia M, Walker JM, Meier H, Frenzel J. Smart Materials and Structures 23, 104002 (2014).

[Saedi, 2016] Saedi S, Turabi AS, Andani MT, Haberland C, Elahinia M, Karaca H. Smart materials and structures, 25, 035005 (2016).

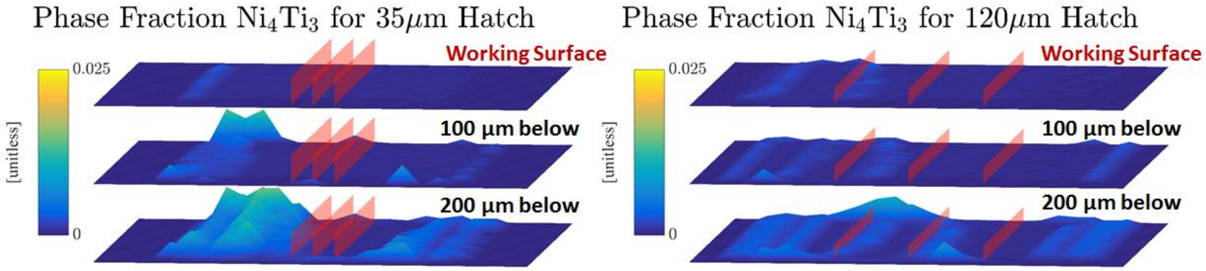

Figure S2: Simulated volume fraction of the Ni_4Ti_3 precipitates at various depths beneath the working surface as a result of three laser scan tracks as indicated: significant precipitation formation begins at roughly 100 µm, or 3 build layers beneath the surface. At this depth, the sample is reheated to a sufficiently high temperature to allow precipitate formation and growth, but not too high where melting occurs. A larger volume fraction of precipitates is found in the 35 µm hatch distance case compared to the 120 µm hatch distance sample because the closer proximity of the laser tracks allows for a prolonged exposure at temperatures where precipitation could occur.

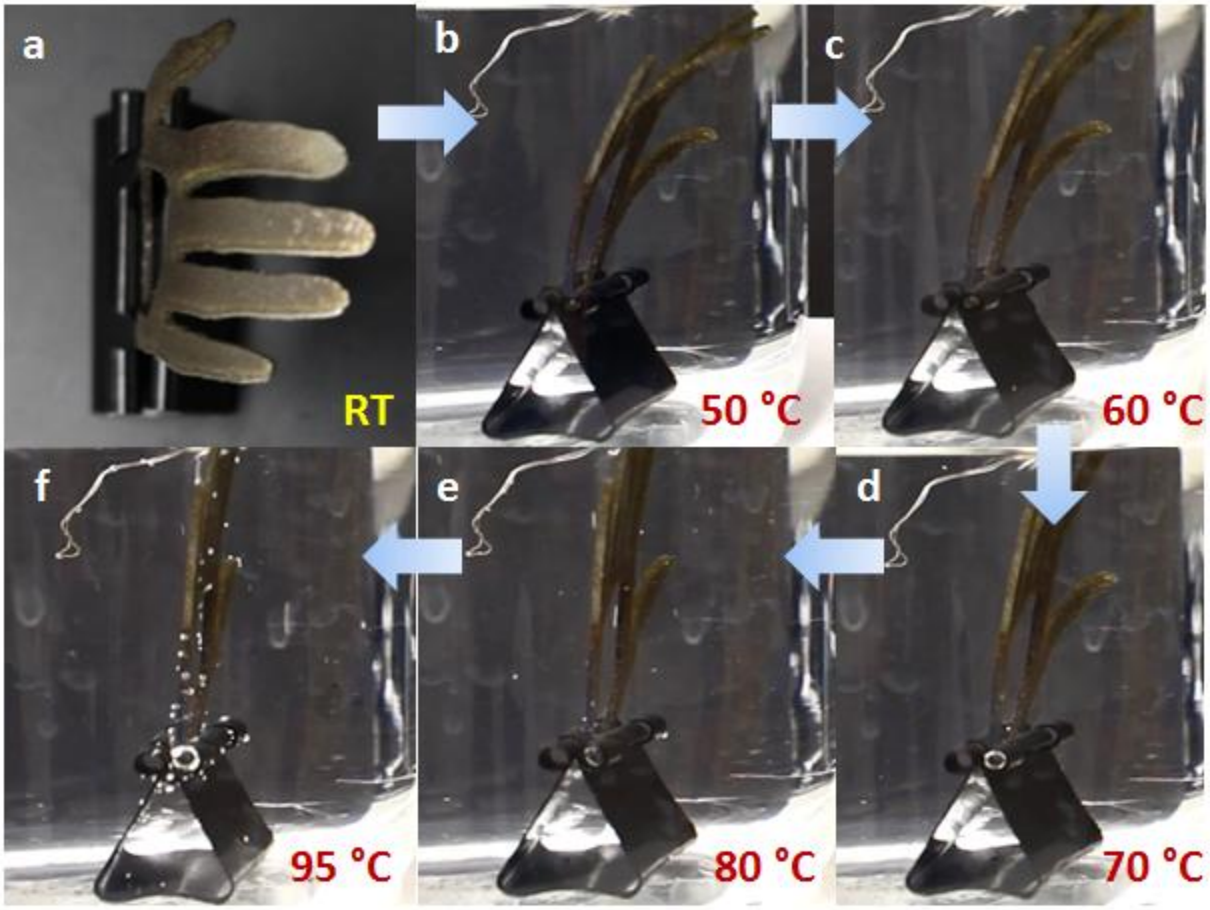

Figure S3: Using the techniques developed to control the location dependent properties, complex active components with multiple locations showing distinct functional response can be created. This example shows a hand shape made from Ni-rich NiTi shape memory alloy where each individual finger has a distinct transformation temperature, and thus activates shape recovery at different times during heating.
